# Investigating the Cytotoxic Effects of *Artemisia absinthium* Extract on Oral Carcinoma Cell Line

**DOI:** 10.3390/biomedicines12122674

**Published:** 2024-11-24

**Authors:** Ioannis Tsamesidis, Aliki Papadimitriou-Tsantarliotou, Athanasios Christodoulou, Dionysia Amanatidou, Chrysostomos Avgeros, Evangelia Stalika, Maria Bousnaki, Georgia Michailidou, Anastasia Beketova, Phaedra Eleftheriou, Dimitrios N. Bikiaris, Ioannis S. Vizirianakis, Eleana Kontonasaki

**Affiliations:** 1 Department of Prosthodontics, School of Dentistry, Faculty of Health Sciences, Aristotle University of Thessaloniki, GR-54124 Thessaloniki, Greece; thanosxristodoulou5@gmail.com (A.C.); evangelia.stalika@gmail.com (E.S.); mbousnaki2689@hotmail.gr (M.B.); anastasiabeketova@yahoo.com (A.B.); kont@dent.auth.gr (E.K.); 2 Laboratory of Pharmacology, School of Pharmacy, Aristotle University of Thessaloniki, GR-54124 Thessaloniki, Greece; alikipapadi@pharm.auth.gr (A.P.-T.); avgerosc@auth.gr (C.A.); ivizir@pharm.auth.gr (I.S.V.); 3 Department of Biomedical Sciences, International Hellenic University, GR-57400 Thessaloniki, Greece; damanatidou@ihu.gr (D.A.); elfther@ihu.gr (P.E.); 4 Laboratory of Polymer and Colors Chemistry and Technology, Department of Chemistry, Aristotle University of Thessaloniki, GR-54124 Thessaloniki, Greece; michailidougeorgia18@gmail.com (G.M.); dbic@chem.auth.gr (D.N.B.); 5 Faculty of Natural Sciences and Technology, Institute of Biomaterials and Bioengineering, Riga Technical University, Pulka 3, LV-1007 Riga, Latvia; 6 Department of Health Sciences, School of Life and Health Sciences, University of Nicosia, 2417 Nicosia, Cyprus

**Keywords:** *Artemisia absinthium* extract, cytotoxic activity periodontal ligament cells, osteogenic differentiation, human tongue squamous carcinoma cell line, artemisinin, phenolics

## Abstract

**Background:** *Artemisia absinthium (A. absinthium)*, commonly known as absinthe, is a perennial plant with distinctive broad ovate pointed leaves of a silvery-gray color, reaching a height of 1.5 m. The utilization of this herb as a source of natural compounds and as the primary ingredient in the alcoholic beverage absinthe has recently seen a resurgence following a period of prohibition. This study investigates the biological effects of *A. absinthium* extract on healthy human periodontal ligament stem cells (hPDLSCs) and the human tongue squamous carcinoma cell line (HSC-3). **Methods:** *A. absinthium* element characterization was performed using High-Performance Liquid Chromatography (HPLC) and the Folin method. Alizarin assays evaluated the osteogenic capacity of human periodontal ligament cells (hPDLSCs) while CCK-8 and MTT determined the cytotoxicity of the extract against HSC-3 and hPDLSCs. **Results:** High artemisinin levels were detected, revealing a concentration of 89 μM (25 μg/mL). The total phenolic concentration of the extract was 1.07 mM +/− 0.11. The in vitro cytotoxicity assays revealed the biocompatible profile of the *Artemisia* extract in hPDLSCs without exhibiting any osteogenic potential. After 24 h of incubation with HSC-3, *Artemisia* extract (10 µM) decreased cancer cell viability by 99% and artemisinin by 64%, and increased the expression of Caspase 3 and 9 almost six and two times, respectively. **Conclusions:** In summary, our preliminary findings suggest that *A. absinthium* extract exhibits a toxic effect against carcinoma cell lines without affecting healthy human periodontal ligament stem cells.

## 1. Introduction

Oral cancer includes various cancer types that can arise in different areas within the mouth. Besides the lips, gingival tissues, and the floor of the mouth, it can also affect the buccal mucosa (the soft lining inside the lips and cheeks) and other regions of the oral cavity [1]. Approximately 90% of oral cancers are oral squamous cell carcinomas, which are more prevalent in men and tend to occur more frequently with advancing age. A major reason for such conditions is attributed to poor oral health, creating an environment in which chronic inflammation, infections, and cellular damage are more likely to occur, all of which promote carcinogenesis. Chronic poor oral hygiene leads to persistent gum disease (periodontitis) and chronic infections that continuously expose the cells in the oral cavity to toxins and inflammatory responses. This ongoing inflammation can lead to cellular mutations and weaken the local immune defense, increasing the likelihood of malignant transformation. These carcinomas develop when cells acquire genetic mutations that lead to the activation of proto-oncogenes or the suppression of tumor-suppressor genes [2,3]. The primary approach for treating oral cancer is surgery, often combined with adjuvant therapies such as radiotherapy or chemotherapy. While many patients achieve positive outcomes with these therapies or anticancer medications, the treatments can be highly toxic to normal cells and tissues. As a result, patients frequently experience a range of side effects, including nausea, vomiting, loss of appetite, diarrhea, and oral mucositis [4,5,6].

Natural compounds derived from herbs have shown strong potential to inhibit the growth of cancer cells while protecting healthy cells from damage [7,8]. The *Artemisia* genus is an excellent example of a natural herb with potential properties against cancer cells. In detail, the *Artemisia* genus belongs to the Asteraceae family, includes around 600 species found worldwide (except Antarctica), predominantly in the Northern Hemisphere, and thrives across diverse habitats, from arid zones to wetlands. Known for their ecological adaptability, many *Artemisia* species have significant economic value, being used in traditional medicine and agriculture, with some species serving as widespread, landscape-defining plants [9]. Traditionally, *A. absinthium* has been valued for its diverse medicinal uses, including as a treatment for spasms, fever, digestive issues, and heart stimulation. It has also been used to expel parasites, aid memory, support mental clarity, and reduce liver inflammation [10,11]. The bioactive constituents of *A. absinthium* include sesquiterpene lactones, such as artemisinin and absinthin, and terpenoids, including trans-thujone, γ-terpinene, 1,4-terpeniol, myrcene, bornyl acetate, cadinene, camphene, trans-sabinyl acetate, guaiazulene, and flavonoids, such as quercetin and rutin [12]. It is of great interest that the literature suggests that *Artemisia* species in vitro have shown strong anticancer activity. In detail, the potential anticancer activity of the *Artemisia* species was explored as a result of artemisinin for a number of tumor types, including breast cancer, prostate cancer, ovarian cancer, pancreatic cancer, and lung cancer [13,14]. *Artemisia* extracts inhibit cell growth in the context of their anticancer effect. Evidence from the literature indicates that several mechanisms are involved in the antiproliferative action by regulating transcription of various target cells through the activation of ERa-related mechanisms [15] and induction of apoptosis in different cell lines by activating caspases, depolarizing the mitochondrial membrane potential, and reducing BCL-2 expression or stopping the cell cycle [16]. In the above context, P53 increases the transcription and expression of genes that cause cell death, like BAX. The BCL2L4 protein, which the BAX gene produces, heterodimers with other BCL2 family members and leads to apoptosis through changes in the mitochondria. *Artemisia* extracts influence the downregulation of antiapoptotic genes, such as BCL2, further regulating cell division. More specifically, it controls cell division by affecting the decrease in the expression of antiapoptotic genes like BCL2, through which tumor growth can be caused, by accumulating the P53-related mutations in a tissue or organ. Following apoptotic gene expression, the permeability of the mitochondrial membrane results in binding a set of factors, which activates Caspase 9, and then Caspase 3, 6, and 7 in a cascading manner [16]. In another study, Wei et al. [17] discovered that the extract derived from *A. absinthium* hindered the proliferation of hepatoma cells by triggering apoptosis, potentially facilitated by endoplasmic reticulum stress and the mitochondria-dependent pathway. Artemisinin’s remarkable flexibility, from its groundbreaking role in malaria treatment to its potential for cancer therapy, antimicrobial activity, antiviral and immunoregulating properties, and even neuroprotection, highlights its multifaceted and promising potential for a wide range of health-related applications and research. Mughees et al. [18] demonstrated that *A. absinthium* extract has the capacity to interfere with cytoskeletal dynamics, disrupt cell cycle regulators, and impair cell–cell interactions, as well as affect intracellular trafficking, cell polarization, and migration in cancer cells, regardless of the breast cancer subtype. These effects highlight artemisinin’s broad potential to disrupt multiple cellular processes essential for cancer cell growth and metastasis. Another study [19] supports the role of reactive oxygen species’ (ROS) accumulation in mediating apoptosis, which parallels artemisinin’s capacity to induce ROS-driven oxidative stress, contributing to cell death in cancer cells, and to act as an antimalarial drug [20]. Together, these findings underscore how *A. absinthium* flavonoids and artemisinin may employ complementary mechanisms to inhibit cancer cell proliferation and induce apoptosis through targeted pathway modulation.

Many plant-based compounds have been studied for their anticancer properties, but not all can selectively target cancer cells without harming healthy cells [21]. The present study aims to assess *A. absinthium* extract specifically for its ability to affect different malignant and non-malignant cells in different ways, which is essential for reducing collateral damage in oral tissues. This focus on selective cytotoxicity has the potential to advance safer cancer treatments, especially for sensitive areas like the oral cavity. In addition, there are relatively limited data on the effects of plant-derived compounds within the field of oral oncology. The present study, by focusing specifically on the effects of *A. absinthium* extract on oral cancer cells, may contribute to the development of eco-friendly, biocompatible, and cost-effective alternative medicinal products for oral cancer, which has distinct physiological and treatment challenges. Although *A. absinthium* is traditionally known for its medicinal properties, its potential in oral oncology is underexplored. By bringing attention to this plant, the present work could inspire further exploration of botanicals in cancer treatment, fostering a potentially more sustainable approach to oncology.

Based on current research and the pressing need for new treatments in oral cancer, this study aims to assess the anticancer effects of *A. absinthium* extract specifically on human tongue squamous carcinoma cells (HSC-3). Furthermore, the study investigates the biocompatibility of the extract in healthy human periodontal ligament stem cells (hPDLSCs) to ensure its safety profile. Our work is novel, as it focuses on the investigation of *A. absinthium*’s targeted effects on cancerous cells alongside its impact on healthy oral cells, which is crucial for advancing safer, plant-based therapeutic options in oral oncology.

## 2. Materials and Methods

### 2.1. Artemisia absinthium Extraction

**Figure 1:**
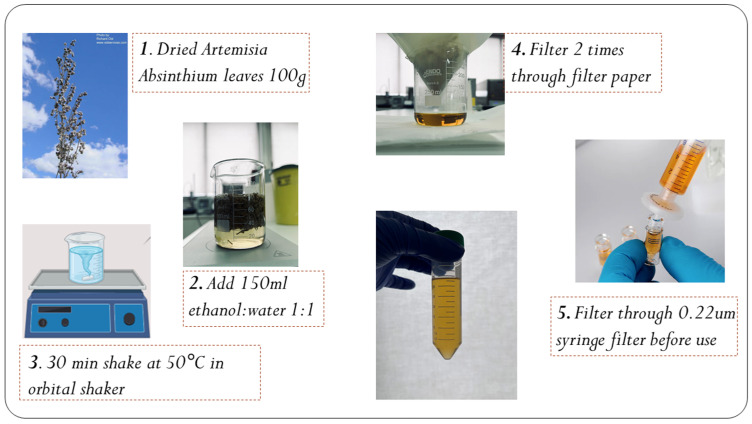
Illustration of the *A. absinthium* extraction.

*A. absinthium* plants were purchased from an accredited plant grower in Ilioupoli, Thessaloniki. The leaves were identified by a botanist from the Department of Chemistry and Pharmacy at the Aristotle University of Thessaloniki, where a comprehensive evaluation was conducted, including morphological analysis, habitat assessment, and sensory examination, to confirm the presence of key characteristics of *A. absinthium*. The aerial parts of the investigated *Artemisia* species were collected during June of 2023 in Crete, Greece (Table 1). The process for the preparation of *A. absinthium* extract is presented in Figure 1. In detail, dried leaves of the plant were washed to remove dust and dirt, dried on paper, then weighed (100 g), manually minced, and extracted (150 mL ethanol: water 1:1) for 30 min at the temperature of 50 °C to avoid possible degradation of bioactive molecules. The extracts were filtered both with filter paper and then through a 0.22 μm syringe. The *A. absinthium* extract was preserved in liquid form at 4 °C in the dark to prevent degradation from light and heat exposure.

### 2.2. Phytochemical Analysis of A. absinthium Extract

#### 2.2.1. High-Pressure Liquid Chromatography (HPLC)

For the extract quantification of artemisinin percentage, acetonitrile HLPC purity (ACN) was supplied from Aldrich Chemicals (Steinheim, Germany). Artemisinin was supplied from Merck, Lyon, France. Quantitative analysis and drug-loading quantitative analysis were performed using a Shimadzu HPLC (Kyoto, Japan) prominence system consisting of a degasser (DGU-20A5, Kyoto, Japan), a liquid chromatograph (LC-20 AD, Kyoto, Japan), an autosampler (SIL-20AC, Kyoto, Japan), a UV/Vis detector (SPD-20A, Kyoto, Japan), and a column oven (CTO-20AC, Kyoto, Japan). For the analysis, the well-established method of Liu et al. was used [22]: CNW Technologies Athena C18, 120 A, 5 μm, 250 mm × 4.6 mm at a column temperature of 30 °C. The mobile phase consisted of H2O/ACN 40/60 *v*/*v*, at a flow rate of 1.0 mL/min. The injection volume was 20 μL and UV detection was performed at 210 nm at 30 °C. The calibration curve was created by diluting a stock methanol solution of 100 ppm artemisinin to concentrations of 0.01, 0.05, 0.25, 0.5, 1.0, 2.5, 5.0, 10.0, 20.0, 30.0, and 50.0 ppm using ultrapure water. All samples including *A. absinthium* extract were filtered (PTFE filters, 0.22 nm pore size) before HPLC analysis.

#### 2.2.2. Total Phenolic Compounds Quantification

Total phenolics were determined colorimetrically using the Folin–Ciocalteau reagent as described by Al-Farsi [23]. The mixture of 200 μL extract and 1.5 mL of Folin–Ciocalteau reagent was incubated at 22 °C for 5 min. Then, 1.5 mL of sodium bicarbonate solution (60 g/L) was added and the mixture was left for 90 min at 22 °C. The absorbance was measured at 765 nm. For the determination of total phenolics, a standard curve of the polyphenol, gallic acid (0.011–0.340 mg/mL or 0.063–2.000 mM), was created. The total phenolics are expressed as mg or mmoles of gallic acid equivalents (GAE) at 100 g of dry plant. Measurements were performed in triplicate and their mean value ± SD is presented.

### 2.3. Establishment of Primary Cultures

Human biopsies of periodontal ligament tissues from a healthy donor, collected during a regular third molar extraction, were used to create hPDLCs cultures. In tissue culture flasks containing 5 mL of DMEM, 10% fetal bovine serum (FBS, Invitrogen, Waltham, MA, USA), and antibiotics (100 U/mL medium of penicillin, 100 mg/mL streptomycin, Invitrogen), small fragments of tissues created by mincing were deposited. The cultures were preserved at 37 °C in an incubator with a 95% humidity and 5% CO_2_ atmosphere. After obtaining a significant fibroblast expansion (80% confluence), the cells were trypsinized with 0.25% trypsin/1 mM EDTA and were then cultivated in 24-well plates under normal conditions. hPDLCs had a spindle-like shape and elongated morphology. The Institutional Ethical Committee approved the project (#110/10-2-2021). The phenotype of the hPDLCs was confirmed with flow cytometry already published in previous work [24].

### 2.4. Biocompatibility Assay

The cellular metabolic activity of hPDLCs in contact with *A. absinthium* was evaluated on days 1 and 3. Cells of passage 2 were seeded in 96-well plates (1 × 10^4^ cells/well) and were allowed to attach for 24 h. Cells were then exposed to different amount values of *A. absinthium* extract: 20 μg/mL (0.23 μL), 200 μg/mL (2.3 μL), and 2000 μg/mL (23 μL), which correspond to 0.1 μM, 1 μM, and 10 μM artemisinin in the total extract, respectively for 1 and 3 days of incubation, and experiments were performed in sextuplicate in 200 μL total volume. Evaluation of mitochondrial activity and, thus, cell proliferation was performed and calculated with the MTT assay. The optical density of the final product was determined spectrophotometrically at a wavelength of 570 nm and a reference filter of 630 nm using a microplate reader (Epoch, Biotek, Biotek Instruments, Inc, Winooski, VT, USA). The results are presented as an average percentage with respect to the controls’ values (cells without *A. absinthium* extract).

### 2.5. Osteogenic Activity

The same set of experiments were performed to evaluate the osteogenic differentiation activity of the tested compound. In detail, *A. absinthium* extract (20 μg/mL and 2000 μg/mL) was employed and then seeded with the hPDLCs. In detail, cells with a number of 4 × 10^4^ were seeded onto 12-well plates 24 h before the experiment. Osteogenic medium (OM) was used for the differentiation of hPDLCs, which contained complete culture medium-CCM (α-minimum essential media (α-MEM) (PAN BIOTECHGmbH, Aidenbach, Germany); 10% FBS (BIOWEST, Nuaillé, France); and antibiotics) enhanced with 0.01 µM dexamethasone (Cayman Chemical Company, Ann Arbor, MI, USA); 50 µg/mL L-ascorbic acid 2-phosphate (Cayman Chemical Company, MI, USA); and 10 mM sodium β–glycerophosphate (Cayman Chemical Company, MI, USA). The experiment included the following cell culture groups: cells cultured in OM with the addition of *A. absinthium* extract (1), cells cultured in conventional medium (CCM) with the addition of *A. absinthium* extract (2) cells cultured in optical density (OD) without any addition as control (3) and cells cultured in CCM without any addition as control (4).

The experiment was executed at two time points (21 and 28 days), with the OM and CCM being changed every 2 days. The impact of artemisinin on the osteogenic differentiation of hPDLCs was evaluated through alizarin red staining (ARS).

### 2.6. Anticancer Activity

#### 2.6.1. Cytotoxicity Assay-CCK8

To assess the potential anticancer activity of *A. absinthium* extract, a cytotoxicity evaluation was conducted on the HSC-3 cell line (Human Squamous Tongue Carcinoma cells). The cells were cultured in DMEM (Dulbecco’s modified Eagle Medium) enriched with 10% FBS (fetal bovine serum) and 1% PS (penicillin-streptomycin) while maintained in an incubator under the following conditions: 37 °C and a humidified atmosphere containing 5% *v*/*v* CO_2_. For the evaluation of cytotoxicity, the cells were cultured in a 96-well plate at an initial concentration of 5 × 10^4^ cells/mL and allowed to attach for 20 h before the addition of each compound. The substances tested included the compound artemisinin and the *A. absinthium* extract. The concentrations of *A. absinthium* extract were: 20 μg/mL, 200 μg/mL, and 2000 μg/mL, and of artemisinin compound were 0.1, 10, and 100 μΜ. In detail, artemisinin was dissolved in DMSO (dimethyl sulfoxide). It is worth noting that the concentration of DMSO in the culture was 0.01% *v*/*v*, in which no detectable effect on cell toxicity was observed. To determine the cytotoxicity of each compound, the cells were allowed to grow for 24 h under the effect of each substance. Subsequently, Cell Counting Kit-8 (CCK8, St. Louis, MO, USA, Sigma-Aldrich) reagent was added to each well and the plate was incubated for 4 h at 37 °C. After incubation, the OD for each well was determined at 450 nm in a multifunctional microplate reader. Wells containing only DMEM and the CCK-8 reagent were used as blank control. Data are presented as mean ± standard deviation (SD) of triplicate incubations.

#### 2.6.2. Gene Expression Level-qPCR

For further validation of the potential anticancer activity of *A. absinthium* extract and artemisinin, the gene transcriptional activity of 3 apoptosis and cell cycle biomarkers (Caspase 3, Caspase 9, and Bcl-2) (primers in Supplemental Table S1) was evaluated via real-time PCR with KAPA SYBR^®^ FAST qPCR Master Mix (2X) Kit (Roche, Basel, Switzerland). HSC-3 cells were exposed to the same conditions as in Section 2.6.1. After 24 h, the cells were harvested and rinsed twice with PBS 1X. Total RNA was isolated through Tritidy-G (PanReac Applichem, Darmstadt, Germany). RNA integrity was determined via a 1% agarose gel electrophoresis with ethidium bromide. The quantity and purity of RNA were detected spectrophotometrically with Nanodrop 2000 (ThermoFisher, Waltham, MA, USA). cDNA was synthesized with QuantTect^®^ Reverse Transcription Kit (Qiagen, Valencia, Spain). All data are presented as mean ± standard deviation (SD) of triplicate incubations.

### 2.7. Statistical Analysis

For all the in vitro experiments (biocompatibility, osteogenic assay, and anticancer activity) a statistical t-test and one-way analysis of variance (ANOVA) were performed via the use of the SPSS program, and the significance level was determined at a = 0.05.

The IC^50^ value of the *A. absinthium* extract was estimated from nonlinear regression of the inhibition plot where HSC3 cell activity was plotted against increasing concentrations of the extract.

## 3. Results

### 3.1. Phytochemical Analysis of A. absinthium Extract

#### High-Pressure Liquid Chromatography (HPLC) and Total Phenolic Quantification

The *A. absinthium* extract was quantified by HPLC, confirming the presence of artemisinin at a concentration of 89 μM, which is within the reported literature range. The total phenolic yield was 1.07 ± 0.02 mmol, or 182 ± 3.6 mg GAE per 100 g of dry plant material using the Folin–Ciocalteau reagent as described by Al-Farsi [22].

### 3.2. Biological Evaluation

#### 3.2.1. Biocompatibility Assay

**Figure 2:**
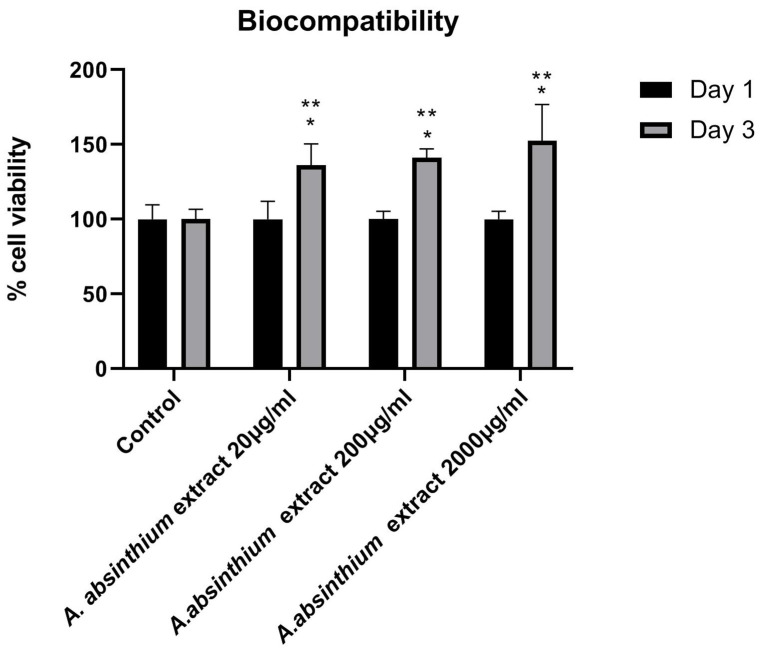
Viability (%) of hPDLC cells after 1 and 3 days of incubation exposed to different concentrations of *A. absinthium* extract (20 μg/mL, 200 μg/mL, and 2000 μg/mL). All values are presented as mean ± standard deviation (SD). The symbol * represents the statistically significant differences between each concentration and the control group. The symbol ** represents the statistically significant differences between days 1 and 3 for each concentration.

The results of the MTT assay show that after 24 h of cell incubation, no statistically significant differences were detected between all the concentrations of substance and positive control, reaching approximately 100%. After 3 days of incubation with *A. absinthium* extract, hPDLCs proliferation was observed in all the tested concentrations compared with the control. Specifically, the highest percentage of cell proliferation (150%) was detected for the concentration of 2000 μg/mL, however, without statistically significant differences with the other two concentrations (Figure 2).

#### 3.2.2. Osteogenic Activity

**Figure 3:**
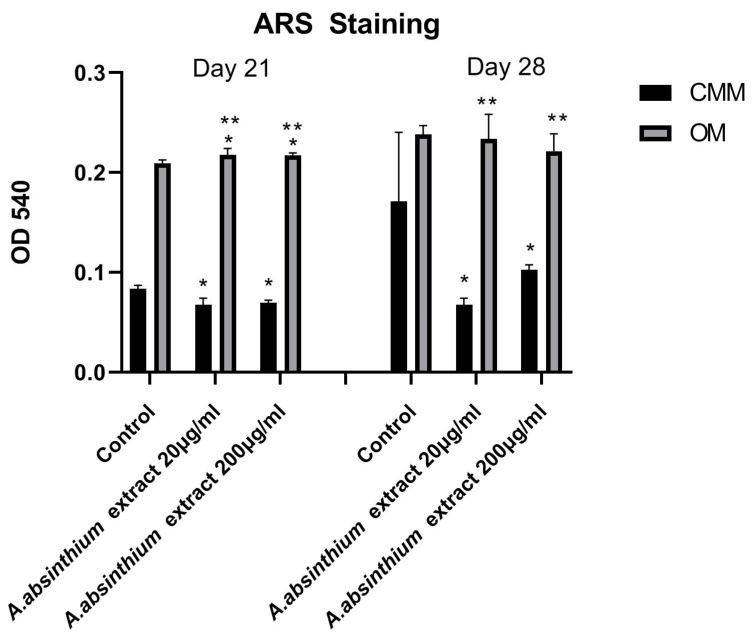
Quantitative analysis of Alizarine straining (ARS) in hPDLCs cultured in CCM and OM media in the presence of *A. absinthium* extract (20 μg/mL and 2000 μg/mL). All values are presented as mean ± standard deviation (SD). The symbol * represents the statistically significant differences between each concentration and the positive control group, while the symbol ** represents the statistically significant differences between conventional the medium (CMM) and osteogenic medium (OM) for each concentration.

After 21 days of incubation, statistically significant differences between cells grown in OM and CCM were observed in all tested groups presented in Figure 3. On the other hand, no statistically significant differences were observed between high (2000 μg/mL) and low (20 μg/mL) *A. absinthium* extract amounts and the control for cells grown in OM medium. After 28 days, the highest concentrations of ARS were observed in the control group for both culture media. Furthermore, the ARS optical density for the two tested groups presented no statistically significant differences compared to the positive control group, suggesting that *A. absinthium* extract does not suppress biomineralization of hPDLCs even at the highest tested concentrations.

#### 3.2.3. Anticancer Activity

##### Cytotoxicity Assay-CCK8

**Figure 4:**
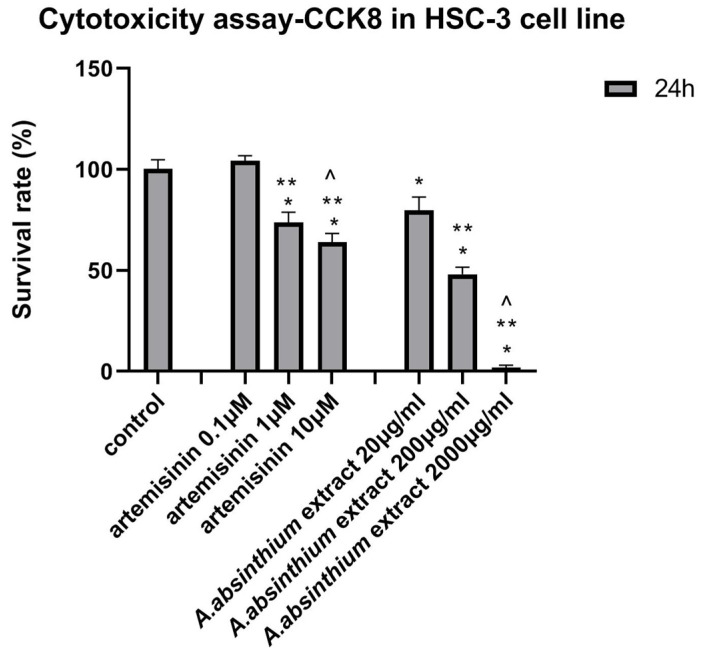
Survival rate (%) of HSC-3 cells after 24 h incubation under the effect of artemisinin (0.1 μM, 1 μM, and 10 μM) and *A. absinthium* extract (20 μg/mL, 200 μg/mL, and 2000 μg/mL). All values are presented as mean ± standard deviation (SD) of triplicate incubations. The symbol * represents the statistically significant difference, at a = 0.05, between each concentration and the control group. The symbol ** represents the statistically significant difference between each concentration examined and the lowest concentration for each substance. Finally, the symbol ^ represents the statistically significant difference between the higher concentration and the intermediate concentration. The statistical tests used for this analysis were one-way ANOVA and t-tests, with a significance level of *p* < 0.05.

**Figure 5:**
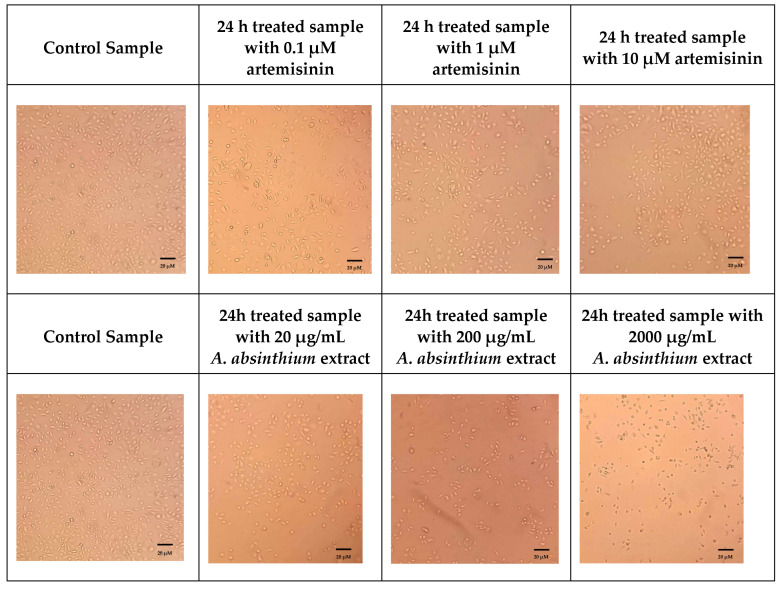
Microscopy images of HSC-3 oral squamous cell carcinoma cells under 10× magnification following 24 h incubation with artemisinin (0.1 μM, 1 μM, and 10 μM) and *A. absinthium* extract (20 μg/mL, 200 μg/mL and 2000 μg/mL). Control (untreated) HSC-3 cells are also shown for comparison.

**Figure 6:**
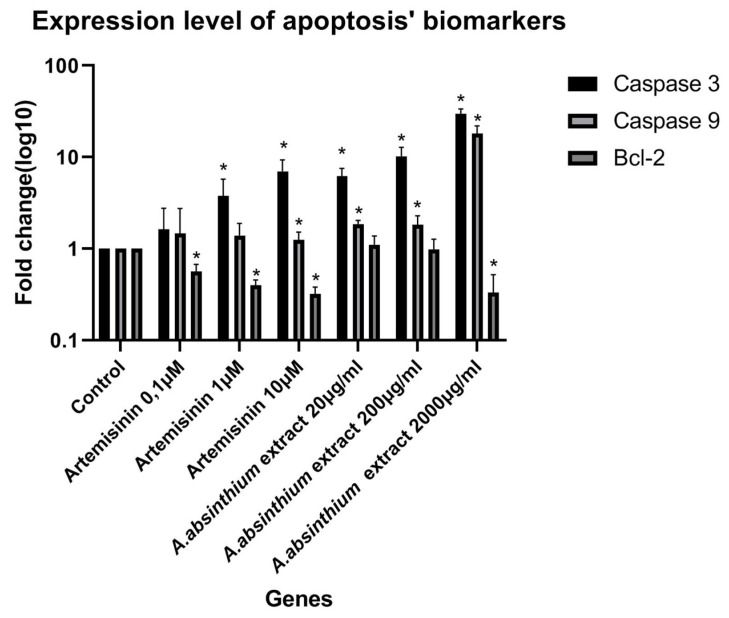
Expression level of apoptosis biomarkers in HSC-3 cells after 24 h incubation under the effect of artemisinin (0.1 μM, 1 μM, and 10 μM) and *A. absinthium* extract (20 μg/mL, 200 μg/mL, and 2000 μg/mL). All values are presented as mean ± standard deviation (SD) of triplicate incubations. The symbol * represents the statistically significant difference between each concentration and the control group. The statistical tests used for this analysis were one-way ANOVA and t-tests, with a significance level of *p* < 0.05.

Based on Figure 4 and Figure 5, it is evident that the presence of artemisinin and *A. absinthium* extract has a significant impact on the growth of HSC-3 cells. Specifically, the bar graph demonstrates that within a 24-h timeframe, a concentration of 10 µM of artemisinin resulted in a 64% reduction in cell growth (Figure 4) and increased the expression of Caspase 3 and 9 almost six and two times, respectively (Figure 6). Furthermore, a statistically significant reduction of Bcl-2 expression was observed (Figure 6). Similarly, the extract at 2000 μg/mL exhibited a remarkable 99% decrease in viability of HSC-3 cells (Figure 4). The latter is further supported by the microscopic images, which reveal morphological changes and affected cell distribution in the plate (Figure 5). Moreover, the expression of Caspase 3 and 9 was increased more than 10 times compared to control cells, while the expression level of Bcl-2 was reduced by over 50%. The IC_50_ of artemisinin, according to the dose-dependent results in HSC-3 cells, is 1.83 μΜ, and of *A. absinthium* extract 134.29 μg/mL.

## 4. Discussion

The findings of our study highlight the biocompatibility of *A. absinthium* extract in hPDLCs, reinforcing its potential as a natural, non-toxic agent for biomedical applications. Furthermore, according to Nair et al. [25], the *Artemisia* species contain varying levels of artemisinin, typically ranging from 25 to 100 μg/mL. Our extract falls within this reported range, supporting its consistency with naturally occurring artemisinin concentrations observed in similar studies without toxic effects. This alignment reinforces the relevance of our extract’s potency and provides a reliable basis for comparison in therapeutic applications. The MTT assay results demonstrated that all tested concentrations of the extract maintain cell viability around 100% after 24 h, suggesting that the extract does not exert cytotoxic effects on hPDLCs, even at higher concentrations. hPDLCs were selected as the normal cell line for our study due to their critical role in maintaining oral health and tissue integrity. As integral components of the periodontal ligament, hPDLCs are among the first cells in the oral cavity to encounter harmful inhaled or ingested substances. They are essential for preserving periodontal health by supporting tooth structure, facilitating tissue repair, and modulating inflammatory responses. Additionally, their involvement in tissue regeneration and response to external stressors makes them an ideal model for studying cellular reactions to potentially carcinogenic compounds within the oral environment [26]. In our study, the immediate lack of toxicity in hPDLCs is a significant advantage, indicating that *A. absinthium* can be safely integrated into cellular environments without compromising cell health. Furthermore, the effect of *A. absinthium* extract, evaluated in recent studies, suggests that artemisinin compounds can be effective drugs for osteoclast-related bone diseases, including osteoporosis and osteoarthritis. The role of artemisinin derivatives against bone resorption has been demonstrated in various animal models. These models include osteoporosis models in ovariectomized mice [27,28], LPS-induced and Ti-particle-induced osteolysis mouse models [29,30], and osteoarthritis-induced bone loss models [31,32]. Artemisinin compounds (dihydroartemisinin, artesunate, artemether) inhibit bone resorption by suppressing RANKL-induced osteoclast differentiation through multiple signaling pathways [30]. Furthermore, studies have shown that artemisinin has osteoprotective and osteostimulating effects on various progenitor cells, including human mesenchymal stem cells (HMSCs) [33] and dental pulp stem cells (DPSC) [34]. These findings suggest that artemisinin may have potential applications in fracture treatment and tissue engineering. Moreover, it was found that artemisinin activated the Wnt/β-catenin signaling pathway and upregulated CA9 expression in DPSCs, which restored osteogenic differentiation in DPSCs under inflammation and hypoxia conditions [34]. Fang et al. reported that artemisinin activates the c-Raf-Erk1/2-p90rsk-CREB signaling pathway and protects BMSCs from oxidative stress injury in a dose-dependent manner [35]. A possible explanation for not observing higher biomineralization in the present study could be the presence of various bioactive compounds in the extract, such as phenolics, that block the reactive oxygen species (ROS)-mediator pathways. Our results on phenolic concentrations are comparable to the ones obtained by Mohd Yasin Bhat [36]. They are relatively low and are similar to the total phenolic concentration of first-year vegetation [37]. Higher concentrations are found in older plants [36]. Among the factors that affect the phenolic content are the years of vegetation, growing region, harvest time, extraction method, etc. [37,38,39]. In detail, some phenolic compounds can generate ROS when oxidized. ROS can influence cellular processes, including those involved in biomineralization. High levels of ROS can disrupt cellular functions and affect the synthesis and deposition of mineralized tissues [40]. Various factors have been recognized to promote the transition of antioxidant phenolic compounds into ROS-generating agents. The structure of phenolics, and in particular the number of their hydroxyl groups, increases the possibility of their action as prooxidants [41,42]. To summarize, the results of the alizarine assay suggest that although a potential restriction of biomineralization could have taken place due to the presence of phenolics, a balancing effect was observed, probably due to artemisinin and its proactive role in osteoblastogenesis. Additionally, Caspase 3 and 9 expression was elevated more than tenfold compared to control cells, while Bcl-2 expression levels were reduced by over 50%. This modification on the transcription level of genes that are connected with apoptosis and cell cycle arrest may indicate that both artemisinin and *A. absinthium* extract lead HSC-3 cells to death through apoptosis pathways. Notably, even at the lowest concentration tested (0.1 µM), the extract exhibited a substantial impact on cell survival, reducing it by 80%. Comparable studies conducted on the YD-10B (human oral squamous cell carcinoma cell line) determined a cytotoxic activity of the extract at 100 µM after 72 h of incubation through an MTT assay [43]. Interestingly, the same study demonstrated that certain derivatives of artemisinin, such as deoxoartemisinin, exhibited a 50% reduction in cell viability at a concentration of 6 µM over the same time period [42]. It is noteworthy that some of artemisinin’s derivatives exhibit approximately fivefold higher anticancer activity in oral cancer cells compared to known drugs, such as cisplatin and paclitaxel [44]. Additionally, another study utilizing the TUNEL assay on the IHOK cell line (HPV 16 immortalized/transformed human oral epithelial cells) revealed that artemisinin increased the cell death rate through apoptosis at an optimal concentration of 400 μM over a 48-h exposure [45]. Furthermore, a more recent study conducted on a xenograft nude mouse model of Oral Squamous Cell Carcinoma (OSCC) showed that artemisinin could inhibit cell growth in a range of concentrations starting from 50 μΜ [46]. To the best of our knowledge, no specific study has demonstrated the extract effects in oral cancer cells. However, several anticancer studies have examined its possible anticancer properties on other types of cancer cells, particularly on liver carcinoma. More specifically, the cytotoxic effect of the extract at 1 mg/mL was examined after 72 h of incubation in the Huh-7 liver cancer cell line compared to Vero cells (monkey kidney epithelial cells) [47]. The results indicated a significant reduction in the viability of cancer cells, while normal cells remained unaffected. The findings of other studies have corroborated similar results [48]. These aforementioned outcomes underscore the potential anticancer properties of both artemisinin and *A. absinthium* extract, thereby suggesting their potential pharmacological applications in oral cancer. Furthermore, a pre-clinical toxicity assessment of *A. absinthium* extract-loaded polymeric nanoparticles, evaluating their safety profile and potential adverse effects, revealed the LD50 cut-off value of the extract-loaded polymeric nanoparticles (ANPs) to be 500 mg/kg [49].

## 5. Conclusions

The extract from *A. absinthium* shows significant potential as a treatment for human tongue squamous carcinoma (HSC-3). It effectively enhances the activity of Caspase 3 and 9, inducing cancer cell death, while remaining compatible with healthy human periodontal ligament stem cells. Importantly, this extract that reduces cancer cell viability does not impact the health or osteogenic capabilities of normal periodontal ligament stem cells. Taken together, these results indicate that *A. absinthium* extract can be safely introduced into cellular environments without compromising cell health and holds promise as a potential anticancer agent, particularly for treating tongue squamous carcinoma. Nonetheless, more studies are needed to better understand its mechanisms and to assess its safety and effectiveness in living organisms.

## Figures and Tables

**Table 1:** Information of the *A. absinthium* specimen for verification.

Voucher No.	125
Collection date	14.6.2023
Locality	Crete, Greece
GPS coordinates	35.417416° N, 24.530005° E
Dried plant of *Artemisia absinthium*	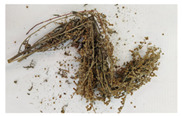

## Data Availability

All data are presented in the manuscript.

## Supplementary Materials

The following supporting information can be downloaded at: https://www.mdpi.com/article/10.3390/biomedicines12122674/s1 Table S1: Primer sequences of Caspase 9, Caspase 3 and Bcl-2 genes.
