# Supplementary material for: Investigating the Cytotoxic Effects of Artemisia absinthium Extract on Oral Carcinoma Cell Line
Source: Biomedicines. 2024 Nov 24;12(12):2674. doi: 10.3390/biomedicines12122674 (PMC11726897; doi:10.3390/biomedicines12122674)
Supplement: Supplementary file 1 [file biomedicines-12-02674-s001.zip › biomedicines-3289841-supplementary.pdf]

Supplemental Table S1. Primer sequences of Caspase 9, Caspase 3 and Bcl-2 genes

Genes	Forward primer sequence	Reverse primer sequence
**Caspase 9**	**5'-TCGAAGCCAACCCTAGAAAA-3'**	**5'- CCTCCAGAACCAATGTCCAC-3'**
**Caspase 3**	**5'-GGTTCATCCAGTCGCTTTGT -3'**	**5'- AATTCTGTTGCCACCTTTCG -3'**
**Bcl-2**	**5'-ACTTCGCCGAGATGTCCA -3'**	**5'-CAAAGAAGGCCACAATCCTC -3'**
